# Genetics and Autoimmunity: Two Sides of the Same Coin or an Epiphenomenon?

**DOI:** 10.31138/mjr.33.1.63

**Published:** 2022-03-31

**Authors:** Rudrarpan Chatterjee, Pankti Mehta, Vikas Agarwal, Latika Gupta

**Affiliations:** 1 Department of Clinical Immunology and Rheumatology, Sanjay Gandhi Post Graduate Institute of Medical Sciences, Lucknow, India,; 2 Department of Rheumatology, Royal Wolverhampton Hospitals NHS Trust, Wolverhampton, United Kingdom,; 3 City Hospital, Sandwell and West Birmingham Hospitals NHS Trust, Birmingham, United Kingdom,; 4 Division of Musculoskeletal and Dermatological Sciences, Centre for Musculoskeletal Research, School of Biological Sciences, The University of Manchester, Manchester, United Kingdom

**Keywords:** inflammatory myopathy, hereditary myopathy, whole genome sequencing, myositis-specific autoantibodies, immunosuppressants, juvenile dermatomyositis, paediatric rheumatology

## Abstract

Distinguishing hereditary forms of myopathy from certain forms of inflammatory myopathy can be challenging. We present 3 cases where a certain degree of overlap was observed between genetics and autoimmunity. A child with juvenile dermatomyositis where heterozygosity for a pathogenic mutation implicated in LGMD1C resulted in a delayed diagnosis. A young lady with anti-SRP positive insidious proximal polymyositis worsening post-partum, diagnosed eventually as LGMD2. An adolescent child referred for proximal myopathy in view of paternal history of LGMD2 but found to have signs of systemic sclerosis with overlap myositis with excellent recovery on therapy. While improvements in whole genome sequencing and detection of myositis specific antibodies have revolutionised the diagnosis and treatment of these diseases, they are still not robust enough and may cloud good clinical judgement in accurate diagnosis and management. Higher sensitivity of these assays is bringing to the fore the possibility that these diagnoses may not be mutually exclusive and might plausibly be concurrent, pending further investigation. These are three interesting cases depicting the difficulties frequently encountered by rheumatologists and neurologists in distinguishing inflammatory from genetic myopathies.

## KEY MESSAGES:

1. Juvenile onset myositis and myositis specific antibodies can co-occur with a genetic background of hereditary myopathies.
2. Laboratory tests and whole exome sequencing should not supersede good clinical judgement.
3. The overlap between genetic mutations and autoimmunity needs further investigation.

## INTRODUCTION

Distinguishing hereditary forms of myopathy from certain forms of inflammatory myopathy can be challenging in the clinic. We present 3 cases where a certain degree of overlap was observed between genetics and autoimmunity.

## CASE 1

### Child with juvenile dermatomyositis (jDM) heterozygous for mutation in the CAV3 gene

An 8-year-old girl was referred to the rheumatology outpatient clinic with a 2-year history of progressive proximal weakness of the lower limb followed by weakness in the upper limbs. Her parents noticed the child’s difficulty in climbing into her school bus to start with, which progressed over a month to inability to get up from squatting position. Subsequently, over the next month, she found it difficult to comb her hair.

She had been seen by the neurology team at the same hospital at the onset of disease 2 years ago. At that juncture, hereditary myopathy was diagnosed in view of proximal muscle weakness predominantly affecting the lower limbs, a waddling gait, Gower’s sign, and calf hypertrophy. Although there was no family history of myopathy, laboratory findings such as creatine phosphokinase levels (CPK, 13000 IU/ml) were supportive of the diagnosis. The possibility of a dystrophy was further substantiated by whole genome sequencing report of heterozygous mutation (Cys72Trp) in exon 2 of the CAV3 (+) gene which was pathogenic for limb girdle muscular dystrophy type 1C (LGMD1C). The parents were counselled regarding the disease prognosis and risks to subsequent pregnancies, and they were subsequently lost to follow-up.

**Figure 1.**
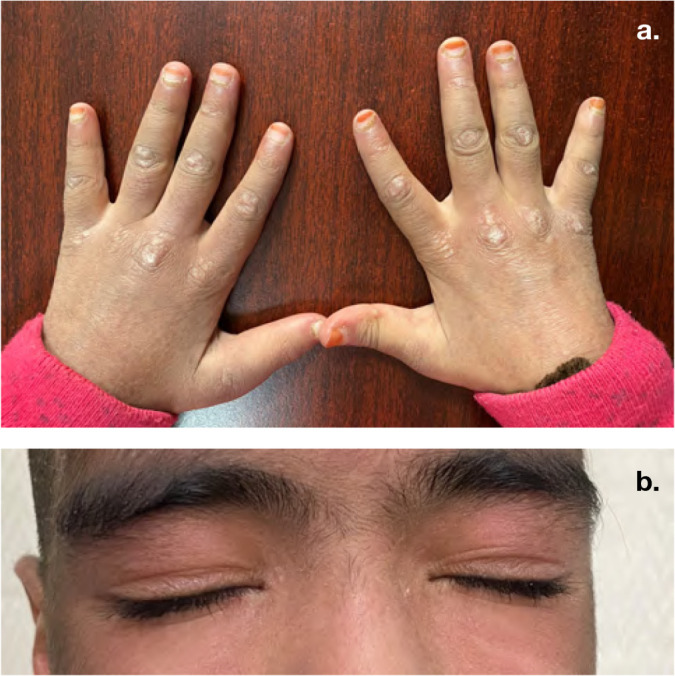
**(a)** Gottron’s papules present on the knuckles of both hands in case 1. **(b)** Erythematous oedema of the eyelids in case 1.

Two years into the illness, a rheumatology consult was sought after she developed a rash on her face for which she had consulted a dermatologist to no avail. On detailed history, we learnt that the rash had been present 2 years prior as well, although it had a waxing-waning course in the absence of immunosuppressant therapy. Presently she found it difficult to get up from the bed without support suggesting that weakness had progressed to involve truncal muscles over the past 1 year. On examination, she had papular eruptions on the knuckles of both hands, elbows, and knees, suggestive of Gottron’s sign (**Figure 1A**), in addition to erythematous oedema of her eyelids (**Figure 1B**) and wasting of proximal muscles including the biceps, the glutei, and the shoulder girdle with winging of the scapula on forward flexion of the arms. Muscle power was 2 of 5 by Medical Research Council (MRC) grade in proximal muscle groups about the shoulder and hip girdle with a maximum power of 4 out of 5 at the distal muscles. Manual Muscle Testing (MMT8) score was 54 out of a possible 80. She seemed to be a definite case of juvenile dermatomyositis, although genome sequencing obfuscated the diagnosis. The extensive muscle wasting is rare in jDM, although long-standing untreated disease could result in such a picture. At the most recent clinic visit, the CPK levels were elevated (1769 IU/ml) and the Anti-Nuclear Antibody test was positive (4+ Homogenous, 1:100). Myositis specific antibodies were positive for anti-Mi2. In view of the elevated CPK, which suggested ongoing muscle damage above and beyond the atrophy, she was given pulses of methylprednisolone for 3 days followed by 1 mg/kg body weight of prednisolone and Methotrexate 15 mg/week. She improved dramatically over the next week, with the patient being able to get up from the bed on her own and improvement in MMT8 to 72/80 over 4 weeks. The rash also responded to immunosuppression with complete resolution on the 2-month follow up visit. At the last outpatient visit, 4 months after initiating therapy, she was on 0.25 mg/kg of prednisone equivalents and 15 mg of Methotrexate. Although the COVID-19 pandemic and subsequent lockdown has precluded further clinic visits, on teleconsultation, she continues to do well at 8 months of follow-up with no skin rash and minimal residual muscle weakness on the same dose of methotrexate and steroids at 0.125mg/kg prednisone equivalent. Even though the rash and the clinical response is supportive of dermatomyositis in this case, our inability to get a muscle biopsy and needle electromyography (EMG) for her are limitations in this case.

## CASE 2

### Young woman with autoantibody positive proximal myopathy positive homozygous for DYSF gene

A 38-year-old woman presented with a 3-year history of insidious onset difficulty in climbing stairs although upper limbs were spared. Power was 4/5 MRC in proximal muscles about the hip girdle with no weakness in the muscles of the shoulder girdle. In view of proximal muscle involvement and a total CPK level of 7273 IU/ml, she underwent a muscle biopsy which showed perifascicular atrophy, perivascular inflammatory infiltrate consisting of both B and T lymphocytes, degenerating as well as regenerating muscle fibres, membrane attack complexes and no evidence of MHC I or II upregulation. Muscle biopsy was reported as inflammatory myopathy likely to be dermatomyositis. Electromyography showed myopathic changes with active denervation. She was treated with pulse methylprednisolone followed by oral prednisolone at 1 mg/kg body weight along with azathioprine as a steroid sparing agent. However, muscle power was static at 3 months following treatment. At this time her electromyography was repeated. It still showed myopathic changes with features of active denervation. Myositis specific antibodies were positive for signal recognition peptide (SRP) by immunoblot assay (1+) raising a possibility of necrotising myopathy (NM), although immunofluorescence did not show the typical cytoplasmic staining seen with these antibodies. It was hence decided to initiate her on intravenous immunoglobulin therapy, which she received in a total dose of 125g over 5 days, following which, some initial improvement in power was felt by the patient, although there was no objective improvement on examination.

At this juncture, she was seen in the rheumatology clinic and upon detailed history it was learnt that the weakness involved only the lower limb with proximal dominance in the first 2 years of her illness with slow progression, and subsequent worsening was noted three months post-partum when upper limbs got involved. Following the initial subjective improvement, she had had progressive weakness of the upper limb and steady worsening of the lower limb weakness this year. Her CPK levels continued to be high at 4285 IU/ml. The odd features in her case were the predominantly lower limb weakness even 3 years into the disease course and non-response to a wide range of immunosuppression. Although anti-SRP antibodies are fairly specific, such a presentation is not typical. However, weakness dominating in legs more than arms has been described.^1^ Suzuki et al. found a chronic form of myopathy in 5% with anti-SRP antibodies, in line with a previous report by Dimitri et al.^2–3^ Patients with anti-SRP and a chronic presentation tend to be younger, and with poorer response to therapy.^2^

Her MSA were repeated and turned out to be negative this time. As MSA is considered to be highly specific, this was a source of confusion. However, there is a possibility that this was a result of therapy with IVIg. Valiyil et al. have described fall in anti-SRP using the semiquantitative technique of immunoprecipitation and Beneviste et al. using the laser bead assay.^4–5^ However, follow-up titres using line immunoassay after IVIg are not known. In view of the lack of response to a wide range of immunosuppression and predominant lower limb weakness, a whole genome sequencing was done for hereditary myopathies and turned out positive for homozygous affection of DYSF gene exon 52 which was pathogenic for LGMD2/Miyoshi type 1. Her immunosuppression was tapered gradually, and she was referred to neurology after explaining the prognosis of the disease. Eight months post-diagnosis, her disease has continued to follow an indolent course with no improvement in the muscle weakness.

## CASE 3

### Adolescent girl child of a patient with muscle dystrophy presenting with Pm-Scl positive overlap myositis

A 15-year-old girl was referred with a 1-year history of difficulty climbing the stairs at her school which had progressed over the last 3 months to involve upper limb muscles and truncal weakness for the past month, due to which she was presently confined to the bed.

Her father had developed a similar proximal myopathy at the age of 28 years with predominantly lower limb weakness around the hip girdle followed by weakness about the shoulder girdle. However, weakness in him was chronic in nature with slow progression over a decade. Upon evaluation by the neurology team, he was diagnosed as a case of limb girdle muscular dystrophy type 2 with muscle biopsy findings of nuclear internalisation, replacement of large portions of muscle with fibrofatty tissue, and focal areas of inflammatory infiltrate of T and B lymphocytes. Genetic testing was not available at that time. Sixteen years into the illness, he is now wheel-chair-bound and adjusted to disability and rehabilitated vocationally.

The parents believed the child had now manifested similar illness as was told to them during genetic counselling sessions, although the rapidity of progress compared to her father was unusual. On detailed history, she was found to have painful discoloration of fingers, suggestive of Raynaud’s phenomenon for two years, beginning a year prior to the onset of muscle weakness, although it was manifest only during winters. She also complained of inflammatory polyarthralgia involving the small joints of the hands.

On examination, she had sclerodactyly, digital pits, Gottron’s papules, and tightening of the skin over her face. Power in the shoulders was 3/5 MRC and 2/5 in the neck and hip girdle with a composite MMT8 score of 65 out of 80. Her muscle enzymes were elevated with a total CPK of 260 u/L. A clinical diagnosis of overlap inflammatory myopathy with limited scleroderma was made, and subsequently myositis specific antibodies were found positive for PM-Scl. Prednisolone 1mg/kg body weight and methotrexate 15mg/week were administered, with which a rapid improvement in muscle weakness was seen over the first month with MMT8 increasing to 74/80. She has since then had a complete recovery of muscle weakness and arthritis without relapse at 4 months of follow-up while Raynaud’s phenomenon was controlled with cold protection and low dose nifedipine. She subsequently had a flare of arthritis on tapering and stopping steroids at 6 months post her diagnosis. This was controlled by restarting low dose prednisolone at 5 mg per day and she continues to do well till date.

## DISCUSSION

This case series highlights the challenges in the correct diagnosis and distinguishing between idiopathic inflammatory myopathy (IIM) and hereditary myopathies, especially limb girdle muscular dystrophy, oft experienced in the clinic by rheumatologist and neurologists alike. Predominant proximal muscle involvement, subacute course, and elevated muscle enzymes through the course of disease are seen in IIM but also possible in certain inherited forms of muscle dystrophy. Muscle biopsy may not be able to distinguish between the two, especially if there is a degree of chronicity to the illness and atrophy predominates. Perifascicular atrophy and perivascular inflammatory infiltrates consisting of CD4+ T cells, macrophages and dendritic cells are typically seen in dermatomyositis along with complement deposition on the endothelium of blood vessels while endomysial inflammatory cell infiltrates complemented with strong sarcolemmal CD8-MHC I complex expression is more typical of polymyositis.^6^ However, such changes can also be seen in 20–40% cases of limb girdle muscle dystrophy and fascioscapular dystrophy - two important variants which can present in adults and be confused with IIM.^6^ With the wider availability of line immunoblot assays, physicians have taken recourse to testing for myositis specific autoantibodies (MSA) as they are fairly specific (seen in <0.1% of general population) for the diagnosis of IIM. Myoblasts as well as damaged mature muscle fibres can express sequestered antigens and lead to breach of peripheral tolerance. MSA can also be present in any condition that has regenerating muscle fibres due to expression of autoantigens in new muscle fibres. This may explain the presence of anti-SRP antibody in the second patient. It may be logical to assume that muscle degeneration in the context of genetic myopathies could lead to a breach in tolerance and production of autoantibodies. However, in clinical practice, these two entities appear mutually exclusive. These are three interesting cases depicting the difficulties frequently encountered by rheumatologists and neurologists in distinguishing inflammatory from genetic myopathies.

The second case presented here had typical morphological features of IIM on the biopsy, except for the lack of MHC I upregulation. The autoantibody to Signal Recognition Particle (SRP) which is normally seen with necrotizing myositis was also discordant with the inflammatory infiltrate consisting of T and B cells on the biopsy. This highlights the importance of immunohistochemistry staining for MHC I upregulation in the diagnosis of inflammatory myositis. In hindsight, the biopsy features in the second case are more likely to fit in with the dystrophy, rather than necrotising picture seen with anti-SRP antibodies. Our findings are also limited by the unavailability of genetic testing in the parents of both the first and third case. Unfortunately, in a resource-limited setting such as ours, genetic testing is expensive and not accessible to all patients. This further increases the challenge in differentiating between the two conditions at disease onset.

A series of 47 patients with inherited myopathies from the John Hopkins Myositis Centre cohort were screened for myositis specific autoantibodies and two found positive.^7^ A patient of LGMD2A had antibodies to Jo-1 and a patient of facioscapulohumeral dystrophy had antibodies to Mi-2 using the line immunoassay. Both patients had initially responded to immunosuppression and had some features of systemic autoimmunity at the onset. The authors hypothesized that rather than a false positive test, these antibodies could point to a co-existing autoimmune process. Mi-2 and TIF1γ autoantigens have been demonstrated in regenerating muscle fibres in patients of dermatomyositis.^8–9^ These studies did not demonstrate any autoantigens in corresponding areas with no regenerating muscle fibres in the same specimens. Control biopsies from healthy muscle also did not show any antigen exposure. It has also been demonstrated that autoantigen expression correlates with the state of differentiation of the muscle both in vitro and in vivo.^10^ It is possible that in patients with a genetic background of hereditary myopathy, an autoimmune process may be present at the onset of illness due to antibodies directed against antigens exposed in the generating muscle fibres, and this may constitute the second hit that unmasks clinical features of the disease.

In a series of 11 patients with presumed limb girdle muscle dystrophy with no abnormality on genome sequencing, 6 were found to have autoantibodies to HMGCR along with anti-SRP in necrotizing autoimmune myositis. However, there is no such reported literature with anti-SRP antibodies.^11^ Both of these antibodies are seen in necrotising autoimmune myositis. Developing myoblasts in culture have increased autoantigen expression parallel to diseased muscle. It is hypothesised that these regenerating muscle fibres form the source of autoantigens that perpetuates autoimmunity by focusing the immune attack on the regenerating cells themselves. It is possible that anti-SRP antibodies that were present in our case occurred due to a similar mechanism. A possible way to address this question would be to test for a temporal association of MSA positivity in patients with the diagnosis of hereditary myopathy by genetic sequencing. Biorepositories with stored sera could be of use in this regard for sequential MSA tests.

Mutations in Cav3 (caveolin) have been described in patients of LGMD1C with Pro105Leu being the most common.^12^ However, the particular mutation reported in our first case was a Cys72Trp mutation, which, has been reported in a single patient of 82 cases with muscular dystrophies of unknown genetic aetiology.^13^ Of note, heterozygous mutation at the same locus was also detected in a single case in a Brazilian cohort of 100 normal individuals who underwent genome sequencing.^14^ This raises the possibility that this might be a mutation with a low penetrance. It also highlights the fact that genome sequencing, however advanced, should not cloud clinical judgement and classic clinical signs on examination. It is important to be aware of when genome sequencing is warranted in the rheumatology clinic for possible myositis mimics. Slow progression over years, predominantly shoulder or hip girdle involvement and especially differential weakness in the hip extensors disproportionate to flexors on clinical examination should alert us towards a possible hereditary myopathy which needs genome sequencing.^11^ These diagnostic tests are both highly specific as well as minimally invasive, and should occupy a complementary role in the effective classification of disease in the specialities of rheumatology and neurology. We hypothesize that juvenile onset myositis and myositis specific auto-antibodies may co-occur with a background of hereditary myopathy and autoimmunity may be the second hit that brings these patients to the clinic. This is a question that needs to be addressed in future research.
